# Theoretical Explanation for Reduced Body Mass Index and Obesity Rates in *Cannabis* Users

**DOI:** 10.1089/can.2018.0045

**Published:** 2018-12-21

**Authors:** Thomas M. Clark, Jessica M. Jones, Alexis G. Hall, Sara A. Tabner, Rebecca L. Kmiec

**Affiliations:** Department of Biological Sciences, Indiana University South Bend, South Bend, Indiana.

**Keywords:** *Cannabis*, body mass index, obesity, omega-6 fatty acid, theory

## Abstract

**Introduction:** Obesity is treatment-resistant, and is linked with a number of serious, chronic diseases. Adult obesity rates in the United States have tripled since the early 1960s. Recent reviews show that an increased ratio of omega-6 to omega-3 fatty acids contributes to obesity rates by increasing levels of the endocannabinoid signals AEA and 2-AG, overstimulating CB_1_R and leading to increased caloric intake, reduced metabolic rates, and weight gain. *Cannabis*, or THC, also stimulates CB_1_R and increases caloric intake during acute exposures.

**Goals:** To establish the relationship between *Cannabis* use and body mass index, and to provide a theoretical explanation for this relationship.

**Results:** The present meta-analysis reveals significantly reduced body mass index and rates of obesity in *Cannabis* users, in conjunction with increased caloric intake.

**Theoretical explanation:** We provide for the first time a causative explanation for this paradox, in which rapid and long-lasting downregulation of CB_1_R following acute *Cannabis* consumption reduces energy storage and increases metabolic rates, thus reversing the impact on body mass index of elevated dietary omega-6/omega-3 ratios.

## Introduction

The current review and meta-analysis establishes the impact of *Cannabis* use on body mass index (BMI) and obesity rates, and provides a well-supported physiological, causative explanation for this impact. *Cannabis* use appears to reverse the impact of the modern American diet on health by reducing the effects of an elevated ratio of omega-6/omega-3 fatty acids on endocannabinoid (eCB) tone. It is therefore necessary to understand how diet impacts health to understand the health impact of *Cannabis* use.

Diet is the main cause of premature death and disability in the United States. The modern western diet is proinflammatory and obesogenic.^1,2^ Diseases associated with inflammation and obesity include cancer, cardiovascular disease, diabetes mellitus (DM), Alzheimer's disease, mood disorders, autoimmune disorders, liver and kidney disease, and musculoskeletal disabilities.^1–12^ A significant dietary factor contributing to these health problems is an increased ratio of omega-6 (linoleic acid, LA) to omega-3 (α-linolenic acid, ALA) fatty acids,^2,10,13–21^ especially in the context of a high glycemic load and reduced physical activity.

Recent reviews show that dysregulation of the eCB system plays a major role in development of obesity and metabolic disorders, and strongly implicate the elevated omega-6/omega-3 ratio as a primary cause of this dysregulation.^15,18,19,22–29^ Omega-6 fatty acids are precursors of the eCBs *N*-arachidonoylethanolamide (AEA, or anandamide) and 2-arachidonoylglycerol (2-AG). These eCB signals act via receptors, including CB_1_R and CB_2_R, and CB_1_R plays a primary role in energy homeostasis. An elevated dietary omega-6/omega-3 ratio therefore leads to elevated levels of AEA and 2-AG, overstimulation of CB_1_R, and dysregulation of energy homeostasis leading to weight gain.^21–23,25,29–32^

### Metabolic consequences of the modern western diet

Among the defining features of the modern western diet are a superabundance of calories from sugars and refined starches leading to increased glycemic load, and a strongly elevated ratio of omega-6 to omega-3 polyunsaturated fatty acids. The dietary omega-6/omega-3 ratio in hunter-gatherers is estimated to be around 1:1 to 3:1, whereas the ratio in the modern western diet is as high as 20:1 or more.^2,13,16,18,19^ This shift in dietary fatty acids increased sharply as more vegetable oils (especially soybean oil) and grains were incorporated into the diet. Corresponding with these changes in diet, rates of obesity and metabolic syndrome are increasing rapidly.^14^

Obesity is a major health concern, strongly associated with systemic inflammation and metabolic syndrome, with increased risk of DM, a variety of cancer types, cardiovascular disease, autoimmune disorders, anxiety, depression, Alzheimer's disease, and other serious medical conditions.^3,7,8,11,18,33–36^ Dietary dysregulation of the eCB system is emerging as a primary cause of these conditions, suggesting that therapeutic interventions targeting this system should be investigated as a primary way to reduce or eliminate many of the most serious chronic diseases characteristic of modern western societies.

### Overview of the eCB system

The eCB system is a signaling system with a prominent role in homeostasis, and is reviewed extensively elsewhere.^15,22,23,25,26,28,37^ This signaling system occurs within the central nervous system (CNS) and in multiple peripheral organs.

The eCB system involves signals and receptors. The main signals are AEA and 2-AG. A major biosynthetic pathway for each begins with the omega-6 fatty acid (FA), LA, and proceeds through arachidonic acid. From arachidonic acid, multiple pathways and enzymes lead to AEA and 2-AG. AEA and 2-AG act through multiple receptors. Best-known are CB_1_R and CB_2_R, G protein-coupled receptors that are located in the CNS, as well as peripherally on a variety of organs and tissues, including the gut, liver, bones, skeletal muscle, and adipose tissues. The eCB signals AEA and 2-AG are degraded by enzymes, primarily fatty acid amine hydrolase for AEA and other fatty acid ethanolamides, and monoacylglycerol lipase for 2-AG and other monoacylglycerols.^15,22,23,25,26,37–40^

### Impact of the dietary omega-6/omega-3 ratio on the eCB system

**FIG. 1.**
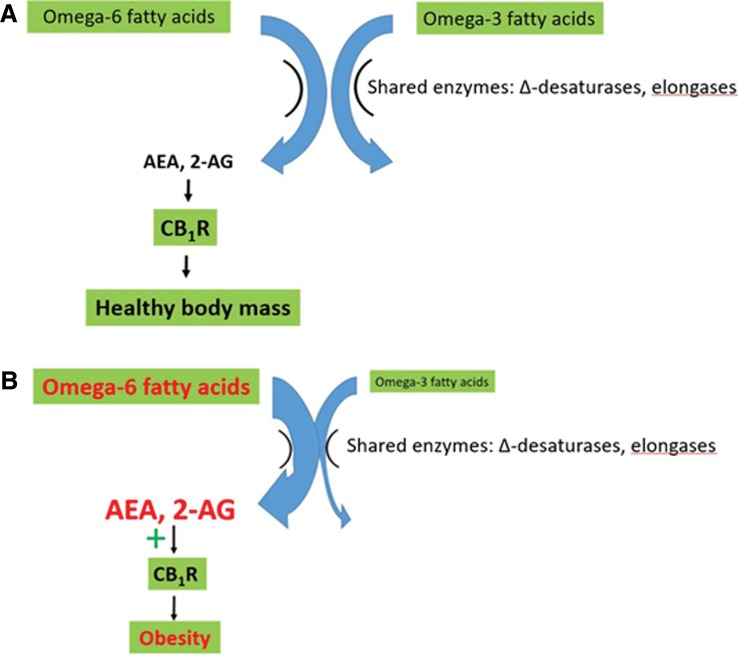
The impact of the modern western diet on the endocannabinoid system. **(A)** In the presence of a natural omega-6/omega-3 ratio, production of the endocannabinoid signals AEA and 2-AG and resulting stimulation of CB1R are compatible with a healthy BMI. **(B)** The modern western diet, with its elevated omega-6/omega-3 ratio, leads to excess production of AEA and 2-AG. This overstimulates CB_1_R, leading to weight gain and metabolic dysregulation. Modified from Freitas et al. (22). AEA, anandamide; *N*-arachidonoylethanolamide; 2-AG, 2-arachidonoylglycerol; BMI, body mass index.

Recent reviews suggest that disruption of the eCB system by an elevated omega-6/omega-3 ratio contributes strongly to the metabolic dysregulation associated with the modern western diet.^15,18,19,22–30,41,42^ Elevated production of the eCBs AEA and 2-AG is central to the health problems associated with the elevated omega-6/omega-3 ratio. Omega-6 FAs are converted to the eCB signals AEA and 2-AG. Therefore, the elevated omega-6/omega-3 ratio results in increased synthesis of AEA and 2-AG, resulting in overstimulation of CB_1_R (Fig. 1). Elevated CB_1_R activity in turn directly causes excess intake, storage, and conservation of energy leading to disruption of body mass and adipose tissue homeostasis.^10,18,19,22,23,25,28–32,41–46^

Omega-3 fatty acids are receiving considerable attention as dietary supplements due to their apparent ability to reduce obesity, inflammation, and associated chronic diseases. Their actions, at least in part, stem from their competition with omega-6 fatty acids for shared enzymes (elongases and Δ desaturases, which are limiting), leading to reduced AEA and 2-AG levels and CB_1_R activity. Because of this competition, it is the ratio between the two groups of fatty acids rather than the absolute amount that is key for energy homeostasis.^4,10,12,15,17–22,47–50^

### Role of eCB and CB_1_R in obesity and metabolic disorders

CB_1_R is a primary mediator of energy uptake, storage, and conservation. It acts to maximize energy uptake and conservation through multiple mechanisms. Stimulation of CB_1_R modulates taste and smell pathways to increase the palatability of food. It stimulates the appetite centers of the brain, leading to hyperphagia and favoring fat accumulation in adipose tissue. At the same time, peripheral eCBs play a major role in regulating appetite, are influenced by the western diet, and AEA reduces energy expenditures, including energy expenditures during sleep.^10,18,22,23,25,28,29,31,32,39,43–46,51–53^.

These actions contribute to homeostasis in the context of a hunter-gatherer diet of plants, plant-feeding animals, and fish. However, the modern industrial western diet, characterized by an elevated omega-6/omega-3 ratio,^16^ leads to chronic overstimulation of CB_1_R.^19,22,23^ When combined with the elevated glycemic load of the modern western diet, this contributes strongly to increased rates of obesity, unfavorable lipid profiles, insulin resistance, exacerbation of inflammation in the liver and kidneys, and increased cardiometabolic risk.^10,29,42,54,55^

The critical role of CB_1_R in accumulation of energy reserves and BMI homeostasis is revealed in studies using CB_1_R antagonists, including rimonabant, as well as the peripherally restricted CB_1_R antagonists URB447 and AM6545. In laboratory and clinical trials, rimonabant was successful at reducing weight, but severe psychiatric side effects, including dizziness, anxiety, depression, and nausea, caused discontinuation of clinical trials.^55,56^

A therapeutic approach that acts both peripherally and centrally on the eCB system but does not cause severe psychiatric side effects is of great interest. Peripherally restricted CB_1_R antagonists such as URB447 and AM6545 are showing promise, as peripheral eCB signaling via CB_1_R plays a key role in stimulation of hyperphagia and dietary fat intake in the context of the western diet.^45,46^ These trials highlight the importance of the eCB system as a target of interest in weight control strategies.^43–46,51,55,56^

The present study summarizes the data on *Cannabis* use, caloric intake, and BMI, establishing conclusively that *Cannabis* use is associated with reduced BMI and obesity rates, despite increased caloric intake. It then provides a theoretical, causative explanation for this paradox. This theory encompasses the causative role in obesity of dietary disruption of the eCB system by an elevated omega-6/omega-3 fatty acid ratio. *Cannabis* (or THC) results in downregulation of CB_1_R, leading to reduced sensitivity to AEA and 2-AG, leading to significant health benefits in the context of this diet.

## Methods

Data on the BMI of *Cannabis* users and nonusers, or studies reporting adjusted odds ratios (AORs) for *Cannabis* users being obese or overweight, were obtained from the literature. Studies addressing the health impact of *Cannabis* use were identified using database searches and citation lists. Studies addressing the impact of therapeutic *Cannabis* use by cancer or AIDS patients or other patients, as a means to increase appetite and caloric intake, were eliminated. Studies in which *Cannabis* was provided to nonusers over a several day period were rejected because short-term weight gain can be caused by water retention from increased sodium intake rather than accumulation of tissue mass. One study^57^ focused on imaging of CB_1_R was rejected due to low sample size (*N*=10 users and *N*=10 nonusers).

The remaining data were compiled into a spreadsheet. Paired *t*-tests were used to compare BMI of users and nonusers and were followed by determination of effect size (Hedges g with bias correction).^58^ For rates of obesity, the mean and 95% confidence intervals of AOR data, χ^2^ test for heterogeneity, and effect size determination using Hedges g were used to compare nonusers with users. When different usage rates were reported, data from the highest dosage group were used in the analysis. The mean across all usage groups, relative to nonusers, is also reported. Caloric intake data from short-term experimental studies were eliminated to ensure that subjects had reached a steady state.

## Results

### BMI data

Nine studies were included that reported BMI of users and nonusers and met selection criteria (Table 1), and an additional two studies were identified that reported lower BMI in *Cannabis* users, but did not provide numerical data. Of these studies, all reported lower values of BMI in *Cannabis* users, and only one of these did not reach statistical significance. A second study did not report statistical analysis of the BMI data. Of those studies reporting significant negative correlations, two reported that longer duration of *Cannabis* use was associated with reduced BMI.^59,60^

**Table 1.** **Published Values of Body Mass Index for *Cannabis* Users and Nonusers**

Reference	Nonuser	Usage pattern	Current user	Current user, highest dosage	*p*-Value or 95% CI
72^a^	28.6 (335)		26.8	**26.8 (451)**	**<0.001**
110	24.4 (23,705)	(women)	23	**23 (6504)**	**<0.05**
	25.4 (14,324)	(men)	24.3	**24.3 (7474)**	**<0.05**
59	28.22 (265)				**<0.05 (joint years)** **<0.009 (dependence)**
		<5 years	26.8 (552)		
		5–10 years	27.1 (42)		
		10–15 years	26.6 (44)		
		15+ years	25.5 (37)	**25.5 (37)**	
60	28 (6667)				
		1–4×/month	24.8 (557)		**<0.001**
		>5×/month	24.1 (326)	**24.1 (326)**	**<0.001**
86	29.1 (2103)		27.2 (579)	**27.2 (579)**	**<0.0001**
70	28.9 (2252)				Not significantly different
		<180 days	28.5 (610)		
		180–1799 days	28.7 (601)		
		>1800 days	28.0 (154)	28 (154)	
71^b^	26.6 (9771)				
		1–4×/month	25 (541)		
		5–10×/month	26.1 (135)		
		11×+/month	24.7 (176)	24.7 (176)	**<0.0001**
78	27 (28)		24 (30)	**24 (30)**	**<0.05**
87	29.1 (2861)		**26.9 (831)**	**26.9 (831)**	**<0.0001**
62	**Numerical data not provided; user BMI < nonuser**				**Not provided**
61	**Numerical data not provided; lower BMI groups contain more*Cannabis*users,*R*^2^=0.96**				**<0.02**
Mean	**27.5 (*N*=60,059)**		**26.0**	**25.5 (*N*=18,272)**	**<0.0005**

Statistically significant differences between *Cannabis* users and nonusers are indicated with bold font.

^a^ Adjusted for age (continuous), gender, small communities (yes/no), more than or equal to secondary school (yes/no), income level (<$20,000, >$20,000, do not know/refuse to answer), marital status (single, married/common law, separated/divorced/widowed), 3.5 h/week of leisure physical activity (yes/no), smoking status (never/former/current smoker with 1–14 cig./day, 15–24 cig./day, 25 cig./day), ever drink alcohol (yes/no/do not know or refuse to answer), total energy intake (kcal/day).

^b^ Effect remained after adjustment for age, gender, education, cigarette smoking, and caloric intake (*p*=0.003).

BMI, body mass index.

**FIG. 2.**
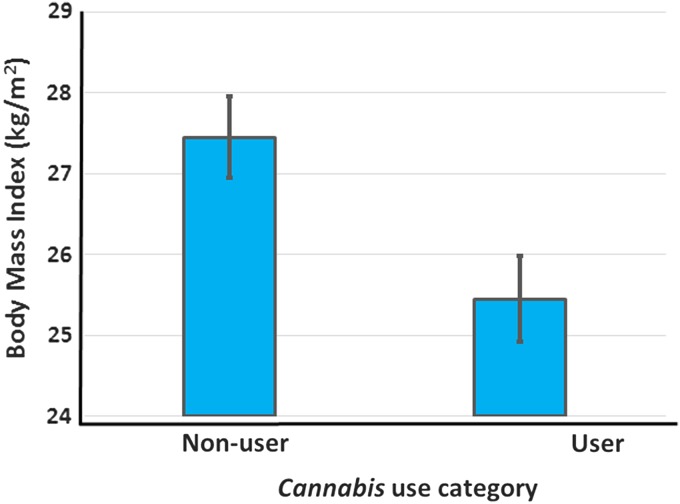
A comparison of BMI (kg/m^2^) of *Cannabis* users and nonusers. Data from current user, highest dosage presented in Table 1. Available data show that nonusers are overweight on average, whereas the mean BMI of users is not different from the upper limit of the healthy weight range. Data are expressed as mean±SEM (*N*=12 data points from 11 studies, *p*<0.001; Hedges g statistic=−1.16).

Across all studies reporting BMI, the overall mean BMI of nonusers was 27.5 kg/m^2^, while that of users (including data for all usage groups) was 26.0 kg/m^2^ (Table 1). Limiting the analysis to the data from the highest dosage or duration of use reported in each study resulted in a mean BMI of users of 25.5 kg/m^2^, a difference of 2 kg/m^2^ that is significantly lower than the BMI of nonusers (*p*<0.001, paired *t*-test, *T*=6.00, Fig. 2 and Table 2). The effect size of *Cannabis* use on BMI is large (Hedges g with bias correction=−1.16)^58^ and the magnitude of the difference in BMI of users and nonusers is of clinical significance. Thus, on average, nonusers in these studies are overweight, whereas *Cannabis* users are significantly leaner and are near the healthy BMI range (18.5–25 kg/m^2^).

**Table 2.** **Published Values for Adjusted Odds Ratios for *Cannabis* Users Being Obese and/or Overweight**

Reference	Usage category	OR users	95% CI	*p*
111^a^				
NESARC, *N*=41,633	1+×/year, <1×/month	0.70	0.63–1.05	**<0.001**
	1×/month–2×/week	0.84	0.62–1.01	
	Daily	**0.61**	**0.46–0.82**	
NCS-R, *N*=9103	1+×/year, <1×/month	0.7	0.44–1.11	**<0.001**
	1×/month–2×/week	0.84	0.54–1.31	
	Daily	0.73	0.43–1.23	
72^b^	Past year	**0.56**	**0.37–0.84**	**<0.05**
63^c^	1×in last month	0.8	0.5–1.2	
*N*=2566	Every few days	**0.5**	**0.3–0.8**	**<0.01**
	Daily	**0.2**	**0.1–0.4**	**<0.001**
88^d^		0.42	0.13–1.36	
65^e^	High vs. low use	**0.2**		**<0.01**
*N*=5141	Sporadic vs. low use	**0.1**		**<0.01**
	Increasing vs low	**1.6**		**<0.05**
112^f^	**Male, past year**			
*N*=40,364	Overweight	0.88	0.67–1.16	
	Obese	0.84	0.6–1.16	
	**Female, past year**			
	Overweight	0.88	0.53–1.45	
	Obese	0.81	0.48–1.38	
**Mean and summary CI**		**0.68**	**0.53–0.84**	**<0.05**

Statistically significant differences between *Cannabis* users and nonusers are indicated with bold font. Only one data point shows AOR >1. Hedges g statistic=−1.07.

^a^ Data from two databases, NESARC, National Epidemiologic Survey on Alcohol and Related Conditions (2001–2002); NCS-R, National Comorbidity Survey–Replication (2001–2003). Adjusted for sex, age, race/ethnicity, educational level, marital status, region, and tobacco smoking status. Prevalence of obesity significantly lower in *Cannabis* users in both data sets (*p*<0.001).

^b^ Age-standardized.

^c^ Odds ratio for BMI ≥25. Adjusted for participant's gender and age, mother's age and education, participant's cigarette smoking, alcohol consumption, anxiety*/*depression and aggression*/*delinquency, participants BMI at 14 years.

^d^ Regular user, OR for abdominal obesity. Adjusted for age, gender, education, participation in at least moderate physical activity, weekly alcohol use, income to poverty ratio, having health insurance, marital status, other illicit drug use and having had rehabilitation.

^e^ Controlled for adolescent obesity status, gender, ethnicity, and average family income.

^f^ Controlled for age, level of education, race/ethnicity, income, marital status, region of country, urban vs. rural residence, and lifetime and past year DSM-IV diagnoses of any mood disorder, any anxiety disorder, any personality disorder, any alcohol use disorder, and nicotine dependence.

AOR, adjusted odds ratio; DSM-IV, Diagnostic and Statistical Manual of Mental Disorders, 4th Edition.

Further support for reduced BMI in *Cannabis* users comes from the study by Warren et al.^61^ Although Warren et al.^61^ did not report BMI values, they grouped obese patients by BMI. The percent of each group that consumed *Cannabis* was negatively and linearly related to the BMI of the group (*R*^2^=0.96). Danielsson et al.^62^ also reported decreased rates of being overweight (BMI >24.9) in *Cannabis* users, but did not provide numerical data for BMI of the two groups. Thus, of 11 studies reporting data on the relationship between *Cannabis* use and BMI, 9 showed a significant negative relationship between *Cannabis* use and BMI while the remaining 2 either reported lower BMI values in *Cannabis* users than nonusers that did not reach statistical significance, or failed to provide statistical analyses (Table 1).

Of course, decreased BMI in *Cannabis* users could result from activities correlated with *Cannabis* use, rather than *Cannabis* use itself. Two of the BMI studies adjusted for potential confounders, and significant differences remained following adjustment (Table 1). Six studies were identified that reported AORs of *Cannabis* users being obese or overweight (Table 2).

Hayatbakhsh et al.^63^ followed a cohort of patients from birth until age 21 and found that subjects who used *Cannabis* showed a strongly reduced incidence of being overweight or obese relative to nonusers. A fully adjusted model that included BMI at age 14 yielded an AOR of 0.2 for daily users being overweight (95% CI=0.1–0.4). BMI was inversely correlated with the frequency of *Cannabis* use, lending support for causation.^63^

Waterreus et al.^64^ found that a significantly lower percentage of users than nonusers were obese (53.7% of nonusers, 36.7% of occasional users, and 28.7% of frequent users were obese; *p*<0.001).

Huang et al.^65^ studied three categories of adolescent *Cannabis* users; high users, sporadic users, and increasing users. Sporadic and high usage groups showed far lower obesity rates than low users (AOR for sporadic use=0.2 and for high use=0.1). In contrast, the subjects on the increasing usage trajectory showed increased obesity rates relative to low users (AOR=1.6). This was the only report identified in the literature of an AOR for obesity >1.

The mean AOR across data points from these studies was 0.68. The effect size was large (Hedges' g with bias correction=−1.074, *N*_cannabis_=18, and *N*_control_=6),^58^ and the mean odds ratio of users across all studies and usage groups (mean OR=0.68) suggests obesity rates are reduced enough in users to provide significant health benefits. Several tests were used to evaluate heterogeneity of the AOR data. The 95% confidence interval of the AOR data of users did not include 1 (95% CI=0.53–0.84). The Wilcoxon rank-sum test using data from the highest usage rates within each study or group resulted in a significant impact of *Cannabis* use on AOR (0.0025 < *p*<0.005; *N*_1_=*N*_2_=9, *U*=9, 72). The χ^2^ test using data from all user groups failed to reject the null hypothesis, however (χ^2^=3.78, 0.1 < *p*<0.05).

A recent review cited Mittleman^66^ as reporting increased obesity rates in *Cannabis* users,^34^ but this appears to be a misinterpretation of the data presented in that study. Mittleman et al.^66^ showed that, of patients who had suffered a myocardial infarction (MI), those who used *Cannabis* were more likely to be obese. This is quite different from finding that *Cannabis* users were more likely to be obese. These data could be interpreted instead as evidence for protection of nonobese *Cannabis* users from MI. These data were therefore not included in the analysis.

Overall, 17 studies have presented data from 19 data sets on the relationship between *Cannabis* use and body mass or rates of obesity. These studies provided a total of 36 individual data points for BMI or AOR, and 35 of these show BMI or obesity values for *Cannabis* users that are less than values for nonusers. Both the BMI data and the AOR data show lower BMI or rates of overweight or obesity in *Cannabis* users (BMI: paired *t*-test *p*<0.001; AOR 95% CI=0.53–0.84) (Tables 1 and 2). Both data sets show strong effect sizes (Hedges g: BMI=−1.16 and obesity AOR=−1.07).^58^

Further evidence comes from the recent observation that legalization of medical *Cannabis* at the state level is associated with a rapid decrease in statewide obesity rates,^67^ and that obese rats exposed to *Cannabis* extract show reduced rates of weight gain.^68^ Indeed, the inverse relationship between obesity and *Cannabis* use in humans led Le Foll et al.^69^ to propose *Cannabis* as a possible therapeutic option for weight loss, and evidence accumulated since then has only strengthened the association.

### Caloric intake data

Interestingly, frequent *Cannabis* users appear to have increased caloric intake relative to nonusers, despite lower BMI.

Rodondi et al.^70^ found that users who had consumed *Cannabis* for more than 1800 days over 15 years consumed on average 619 more calories/day than nonusers, yet showed no difference in BMI (Table 1).

Smit and Crespo^71^ reported lower BMI in users (24.7±0.3) than nonusers (26.6±0.1), despite users consuming 564 additional calories relative to nonusers (*p*<0.0001).

Ngueta et al.^72^ also observed higher values for caloric intake in *Cannabis* users relative to nonusers; although this was not statistically significant (2375 kcal/day vs. 2210 kcal/day; *p*=0.07). Despite this, the users had lower BMI (*p*<0.001).

Foltin et al.^73^ found *Cannabis* users to have a substantial increase (1095 kcal/day) in daily caloric intake, although this was a short-term experimental study rather than a comparison between free-range *Cannabis* users and nonusers.

Across these studies, on average, *Cannabis* users consumed an additional 834 kcal/day relative to nonusers. As BMI of *Cannabis* users is lower than nonusers, this suggests that *Cannabis* users must have increased metabolic rates.

### Previous explanations proposed for lower BMI in *Cannabis* users

Any theory explaining mechanistically how *Cannabis* use causes reduced BMI must consider the paradoxical increase in caloric intake of users. To date, such a theory is lacking and the interactions between *Cannabis* use and obesity are not well understood.^34^

Proposed explanations for reduced BMI in *Cannabis* users include substitution of *Cannabis* for food in brain reward pathways.^61^ Pagotto et al.^74^ suggested that the sedative effects of high doses of *Cannabis* could reduce food consumption, but Rajavashisth et al.^60^ observed detectable effects on BMI at usage rates of four times or less per month (25% of nonusers were obese, whereas 16% of people who used *Cannabis* one to four times/month were obese, *p*<0.001). Sabia et al.^67^ suggested that reduced alcohol use by younger users, and increased physical activity of older users upon initiating medical marijuana use, may be responsible for the observed decrease in BMI.

While all of these factors may contribute, reduced BMI in conjunction with increased caloric intake strongly suggests that the mechanisms causing the observed decreases in BMI or obesity rates of *Cannabis* users must include differences in metabolism, not changes in caloric intake or activity-related energy expenditures alone. These explanations obviously do not account for increased caloric intake in *Cannabis* users. Le Foll et al.^69^ suggested that Δ^9^-tetrahydrocannabinol (THC) may act as a functional antagonist in high eCB tone, as occurs in obesity, reducing BMI in *Cannabis* users.^69^ This is essentially what we are proposing, but does not address the mechanism involved.

### Theoretical explanation for the decreased BMI of *Cannabis* users

There are currently no proposed mechanisms for reduced BMI in *Cannabis* users that account for their increased caloric intake. The central role of CB_1_R in appetite, energy intake, energy conservation, and diet-induced obesity, and the hyperphagia and hypothermia resulting from acute stimulation of CB_1_R by THC, makes CB_1_R a prime suspect for a causative role in the effects of *Cannabis* use on BMI.^22–24,27–32,41–46,74,75^

**FIG. 3.**
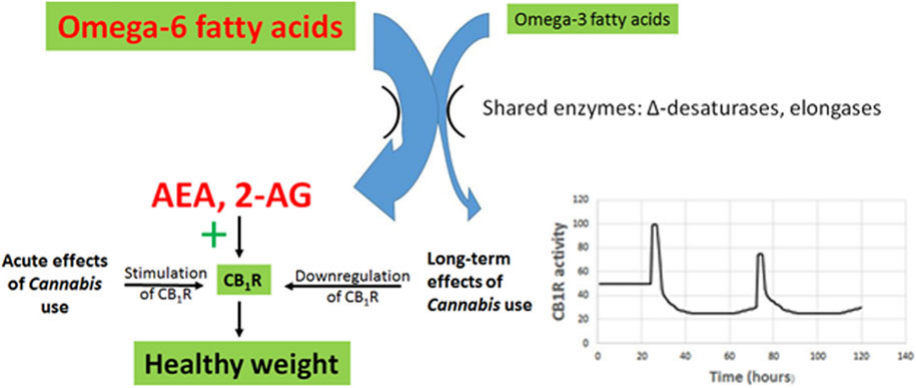
The impact of *Cannabis* use on the endocannabinoid system of people consuming a diet characterized by an elevated omega-6/omega-3 ratio. Acute effects of *Cannabis* and/or THC consumption include hypothermia and hyperphagia, leading to increased energy intake and storage. However, *Cannabis* use also causes long-term downregulation of CB_1_R, leading to decreased CB_1_R activity, as shown in the insert on the lower right, in which each spike follows acute *Cannabis* ingestion, while the overall activity level remains depressed. Decreased CB_1_R activity results in a decrease in energy assimilation and an increase in metabolic rates, resulting in a decline in body mass despite stimulation of CB_1_R during acute exposure. THC, Δ^9^-tetrahydrocannabinol.

A novel theory for the impact of *Cannabis* use on BMI involving changes in CB_1_R expression is proposed here (Fig. 3). This multipart theory includes the following components:

1. A diet characterized by an elevated ratio of omega-6/omega-3 fatty acids, typical of processed foods high in grains and soybean oil, and animals reared on these foods, results in elevated levels of the eCB signals AEA and 2-AG.
  The evidence is well established.^19,22,23,25,28,49^
2. Elevated AEA and 2-AG act to overstimulate the eCB receptor CB_1_R, resulting in increased appetite and palatability of food, increased rates of energy uptake and storage, and decreased resting metabolic rates. These result in dysregulation of glucose and lipid metabolism, metabolic syndrome, and obesity.
  The evidence is well established and is summarized in multiple recent reviews, for example, see Refs.^19,22,23,27–29^
3. Decreased CB_1_R activity reduces obesity and metabolic disruption. Strong evidence in support of this statement is provided in laboratory experiments and clinical trials using CB_1_R antagonists, including rimonabant, AM6545, and URB447.
  Rimonabant caused weight loss, improved lipid profiles, improved glucose sensitivity, and reduced atherosclerosis in animals and human subjects.^55,56,76^ Unfortunately, it also caused severe psychiatric side effects in clinical trials, including depressive disorders, dizziness, nausea, and anxiety, and trials were therefore terminated.^55,56,76^ The peripherally restricted CB_1_R antagonists, AM6545 and URB447, decreased sham feeding of fatty foods and hyperphagia in rats, reducing caloric intake.^45,46^
4. *Cannabis* use causes downregulation of CB_1_R, reducing the impact of enhanced AEA and 2-AG production arising from an elevated dietary omega-6/omega-3 ratio.
  Multiple studies show that CB_1_R is downregulated during *Cannabis* tolerance, and the receptor remains downregulated for about 3–4 weeks after cessation of use.^57,77–80^

### Observations supporting this theory

There is abundant evidence that rates of obesity and metabolic syndrome are increasing with changes in diet.^1–4,6,13,14,16,33,81^

There is abundant evidence that these dietary changes include a shift to a high omega-6/omega-3 ratio.^13–19,22,23^

There is abundant evidence that an elevated omega-6/omega-3 ratio increases eCB tone by increasing AEA and 2-AG levels, overstimulating CB_1_R.^15,18,19,22–30,55,82^

There is abundant evidence that overstimulation of CB_1_R increases adiposity and leads to metabolic syndrome, contributing to chronic diseases.^10,18,19,22,23,25,27–32,41–46,54,55,82^

There is abundant evidence that reduced CB_1_R activity results in weight loss. eCBs are strongly involved in energy expenditures, increasing caloric intake, and reducing whole-body energy metabolism.^24,28,31,32,41–46,48,50,52,55^ The CB_1_R antagonist rimonabant increases O_2_ consumption and resting energy expenditures in both rats and in humans. In rats, it increases O_2_ consumption by 18% at a dosage of 3 mg/kg and 49% at 10 mg/kg after 3 h of exposure. In humans, it increases resting energy expenditures of overweight or obese subjects and leads to weight loss.^55,56^ Similarly, the peripherally restricted CB_1_R antagonists URB447 and AM6545 reduce energy intake. URB447 reduced rates of fat ingestion in sham-feeding rats, while AM6545 attenuated diet-induced hyperphagia.^43,44^

There is abundant evidence that exposure to *Cannabis* and/or THC results in downregulation of CB_1_R. Regular *Cannabis* use is associated with desensitization and downregulation of CB_1_R, and CB_1_R levels remain depressed for 3–4 weeks following cessation of use.^57,77–80^ Because CB_1_R plays a major role in assimilation, storage, and conservation of energy, this downregulation results in decreased eCB tone. According to the theory put forth in this article, acute exposure results in the “munchies,” stimulating appetite and energy consumption and causes hypothermia as metabolic rates decrease. However, rapid downregulation of CB_1_R following consumption leads to long-term effects that more than offset the short-term increase in energy stores that follow acute exposures.

The current meta-analysis provides strong evidence that *Cannabis* use, and/or exposure to THC, results in reduced BMI (Tables 1 and 2 and Fig. 2).

### Predictions arising from theory

#### Prediction 1: *Cannabis* users lose additional weight during abstinence

BMI is reduced in *Cannabis* users, and should decrease even more when users stop using *Cannabis*, because CB_1_R remains downregulated for several weeks following chronic *Cannabis* consumption.^57,77–80^ Recently abstinent users would show reduced appetite and increased metabolic rates during this time. However, they will no longer experience short-term stimulation of appetite, energy intake and storage, and reduced metabolic rates during each episode of acute *Cannabis* consumption. Therefore, weight loss will increase as energy intake and storage remain depressed, and metabolism stimulated, until CB_1_R returns to pre-*Cannabis* use levels.

This prediction is supported, as weight loss during withdrawal from *Cannabis* is one of the seven symptoms of *Cannabis* withdrawal listed in DSM-V.^83,84^

#### Prediction 2: moderate *Cannabis* use reduces the incidence of disorders associated with obesity and metabolic syndrome

Because *Cannabis* use is associated with reduced rates of obesity, it should also reduce rates of obesity-related diseases in users. There is some evidence for this, but results are inconsistent.

Multiple studies, including several using the National Health and Nutrition Examination Survey (NHANES) database, have reported in *Cannabis* users reduced rates of DM, insulin insensitivity, or metabolic syndrome in fully adjusted models, including age.^60,64,72,85–87^ Yankey et al.^88^ also reported decreased DM rates (AOR 0.42) that did not reach statistical significance (95% CI=0.13–1.36). In contrast, analysis of data from the CARDIA data set failed to detect this relationship.^89^ Danielsson et al.^62^ found decreased rates of DM in *Cannabis* users in a dataset of Swedish conscripts (OR 0.74), but unlike the studies from the NHANES data set, this effect was no longer significant after adjustment for age (AOR 0.74 before adjustment, 0.94 after adjustment).

Cannabinoids have potent anticancer properties,^15,90^ and a recent review concluded that *Cannabis* users may have lower rates of cancer than nonusers.^91^ Multiple laboratory studies have shown that THC slows or reverses the progression of Alzheimer's disease, although clinical trials are lacking.^92–96^ In contrast, evidence available to date does not support reduced rates of cardiovascular disease in *Cannabis* users,^97^ although more studies are clearly warranted on this topic.

#### Prediction 3: the occurrence and magnitude of metabolic benefits from *Cannabis* use depend on the dietary omega-6/omega-3 ratio

The impact of diet on the eCB system is predicted to differ among populations because different populations have different diets, consuming different proportions of green vegetables, industrially produced animals, oceanic fishes, and processed foods.

According to the theory established in the current article, populations with diets characterized by a high omega-6/omega-3 ratio will see significantly larger health improvements from *Cannabis* use than those eating diets with more moderate ratios of omega-6/omega-3 FAs. This may explain some of the inconsistencies in the data on the metabolic impact of *Cannabis* use; for example, *Cannabis* use by Swedish populations^62,98^ may not have the same health impacts as *Cannabis* use by Americans due to the different dietary backgrounds and obesity rates of these populations.

*Cannabis* use in the United States appears to provide significant public health benefits due to partial or complete reversal of the metabolic dysregulation caused by the strongly elevated omega-6/omega-3 ratio of the American diet.

#### Prediction 4: *Cannabis* use and omega-3 supplements have similar impacts on health

Both omega-3 FAs and *Cannabis* reduce eCB tone, through distinct mechanisms. Omega-3 FAs compete with omega-6 FAs for the enzymes synthesizing AEA and 2-AG from omega-6 FAs, and omega-3 supplements thereby reduce the synthesis of AEA and 2-AG and reduce stimulation of CB_1_R.^13,21,22,25,49^

*Cannabis* use causes downregulation of CB_1_R,^57,77–80^ reducing the sensitivity to elevated AEA and 2-AG. Thus, the theory predicts that omega-3 FA supplements and *Cannabis* use should have similar positive health impacts in the context of metabolic dysregulation from a diet with an elevated omega-6/omega-3 ratio. However, it is likely that the overlap is not complete as the precursor of AEA and 2-AG, arachidonic acid,^22^ also gives rise to proinflammatory leukotrienes and prostaglandins,^99^ an effect that might not be impacted by decreased CB_1_R tone.

#### Prediction 5: the combination of omega-3 supplements and *Cannabis* or cannabinoids could be a particularly potent treatment for obesity, metabolic syndrome, cancer, and so on

**FIG. 4.**
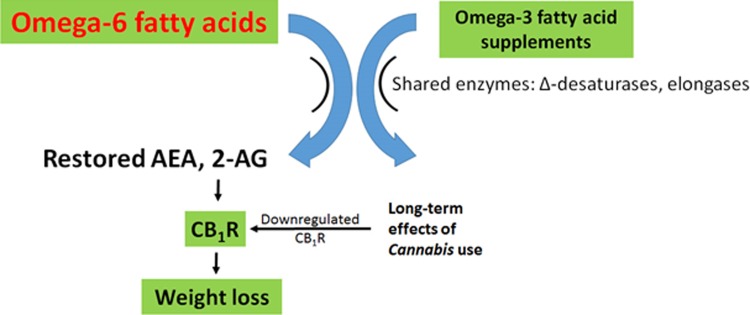
Proposed weight loss therapy based on theory. Daily omega-3 fatty acid supplements (especially with decreased dietary omega-6 fatty acids) will reduce levels of AEA and 2-AG, reducing stimulation of CB_1_R, while weekly *Cannabis* use will cause downregulation of CB_1_R. Thus, this approach will act to both reduce levels of the endocannabinoid signals and reduce the sensitivity of target cells to those signals. The net effect is predicted to be a more potent weight loss strategy than diet alone.

Reducing AEA and 2-AG synthesis with omega-3 supplements, and at the same time reducing CB_1_R density with *Cannabis* use, should reduce BMI and cardiometabolic risk factors more than either option alone (Fig. 4). Note that, because CB_1_R remains downregulated for some time following use, weekly *Cannabis* use may be sufficient to observe significant weight loss and metabolic benefits.

## Conclusions/Summary

Obesity and elevated BMI are strongly associated with disease states, and there are significant financial and public health incentives to develop effective interventions to help people achieve a healthy body mass. Pharmacological weight loss therapy is recommended when BMI is ≥27 in the presence of obesity-related risk factors and >30 in the absence of such risk factors.^53^

The development of pharmacological weight loss methods has been problematic, Rimonabant, a CB_1_R antagonist, showed promise in laboratory studies, but clinical trials were discontinued due to serious psychiatric side effects,^53,54^ although ongoing studies suggest that peripherally restricted CB_1_R antagonists may provide therapeutic benefits in obesity without such psychiatric side effects.^43–46^

Surgical methods such as the lap band or bariatric surgeries are frequently used when dietary or pharmaceutical interventions do not work, and any surgical procedure entails risk and recovery. Surgical procedures are also expensive. Therefore, relatively safe and inexpensive methods to reduce obesity and prevent or reduce some of the most deadly and costly chronic diseases characterizing western societies merit serious consideration.

For many patients, *Cannabis* may be a better option for weight loss than surgery or pharmaceuticals. However, patients with preexisting cardiovascular conditions or prior MIs should avoid cannabinoids or use them with caution.^66,91,100^

A number of states and the federal government have legalized *Cannabis* products containing cannabidiol, but continue to ban legal access to products containing THC. Evidence available at this time suggests that it is ingestion of THC that is responsible for downregulation of CB_1_R, and therefore, for reduced obesity rates of *Cannabis* users. Our theory suggests that the psychoactive effects of CB_1_R stimulation with THC may be a necessary accompaniment to *Cannabis*-induced weight loss, because downregulation of CB_1_R is required for reduced BMI, and it is not yet clear whether microdosing will cause downregulation. However, weekly or biweekly *Cannabis* use may be sufficient as significant decreases in BMI are observed at weekly usage rates.^60^

Medical marijuana use is increasing, leading to decreased use of multiple classes of pharmaceuticals. Patients cite improved symptom management, fewer adverse side effects, and milder withdrawal symptoms as reasons for switching from pharmaceuticals to medical *Cannabis*.^101–108^ Once patients become aware that the side effects of medical *Cannabis* may include weight loss and reduced risk of obesity-associated medical conditions, this shift toward medical *Cannabis* is likely to accelerate. Available data suggest that this will save many lives, not only from reduced rates of obesity-related chronic illnesses but also from reduced deaths from pharmaceutical overdose.^91,105,109^

This study provides a theoretical platform to inform future studies on the correlations between *Cannabis* use and cardiometabolic risk factors. This theory may explain inconsistencies among studies on the impact of *Cannabis* use on metabolic dysregulation, as different populations have different diets. For example, epidemiological studies of the impact of *Cannabis* use by cohorts of Swedish conscripts may reveal different results than epidemiological studies in the United States, due to different levels of obesity in the two countries. Cerdá et al.^98^ found that early, heavy *Cannabis* use among Swedish conscripts is associated with increased mortality later in life. In contrast, Clark^91^ concluded that *Cannabis* use is associated with a substantial decrease in the premature death rate in the United States, as it is associated with reduced rates of cancer, DM, pharmaceutical use, deaths from brain trauma, and may slow the progression of Alzheimer's and other neurodegenerative diseases.

The strong evidence for interactions between the dietary omega-6/omega-3 ratio, obesity, and *Cannabis* use suggests that the balance between positive and negative health impacts of *Cannabis* use will differ in Swedish and United States populations. Evidence suggests that, in the United States, many people may actually achieve net health benefits from moderate *Cannabis* use, due to reduced risk of obesity and associated diseases.

## Abbreviations Used

2-AG: 2-arachidonoylglycerol
AEA: anandamide; *N*-arachidonoylethanolamide
ALA: α-linolenic acid
AOR: adjusted odds ratio
BMI: body mass index
CB_1_R: Cannabinoid receptor 1
CB_2_R: Cannabinoid receptor 2
CNS: central nervous system
DM: diabetes mellitus
DSM-V: Diagnostic and Statistical Manual of Mental Disorders, 5th Edition
DSM-IV: Diagnostic and Statistical Manual of Mental Disorders, 4th Edition
eCB: endocannabinoid
FA: fatty acid
LA: linoleic acid
MI: myocardial infarction
THC: Δ^9^-tetrahydrocannabinol
